# Effect of Trail Bifurcation Asymmetry and Pheromone Presence or Absence on Trail Choice by *Lasius niger* Ants

**DOI:** 10.1111/eth.12248

**Published:** 2014-05-15

**Authors:** Antonia Forster, Tomer J Czaczkes, Emma Warner, Tom Woodall, Emily Martin, Francis L W Ratnieks, M Herberstein

**Affiliations:** * Laboratory of Apiculture & Social Insects, School of Life Sciences, University of SussexBrighton, UK; † Biologie I, Universität Regensburg, UniversitätsstraßeRegensburg, Germany

**Keywords:** *Lasius niger*, asymmetry, pheromone, trail choice, environmental effects, foraging

## Abstract

During foraging, ant workers are known to make use of multiple information sources, such as private information (personal memory) and social information (trail pheromones). Environmental effects on foraging, and how these interact with other information sources, have, however, been little studied. One environmental effect is trail bifurcation asymmetry. Ants forage on branching trail networks and must often decide which branch to take at a junction (bifurcation). This is an important decision, as finding food sources relies on making the correct choices at bifurcations. Bifurcation angle may provide important information when making this choice. We used a Y-maze with a pivoting 90° bifurcation to study trail choice of *Lasius niger* foragers at varying branch asymmetries (0°, [both branches 45° from straight ahead], 30° [branches at 30° and 60° from straight ahead], 45°, 60° and 90° [one branch straight ahead, the other at 90°]). The experiment was carried out either with equal amounts of trail pheromone on both branches of the bifurcation or with pheromone present on only one branch. Our results show that with equal pheromone, trail asymmetry has a significant effect on trail choice. Ants preferentially follow the branch deviating least from straight, and this effect increases as asymmetry increases (47% at 0°, 54% at 30°, 57% at 45°, 66% at 60° and 73% at 90°). However, when pheromone is only present on one branch, the graded effect of asymmetry disappears. Overall, however, there is an effect of asymmetry as the preference of ants for the pheromone-marked branch over the unmarked branch is reduced from 65%, when it is the less deviating branch, to 53%, when it is the more deviating branch. These results demonstrate that trail asymmetry influences ant decision-making at bifurcations and that this information interacts with trail pheromone presence in a non-hierarchical manner.

## Introduction

Ants are central place foragers, and an individual worker can potentially use multiple sources of information when travelling between the nest and a feeding site. Two well-studied types of information are available to foragers: private information, particularly memory, and social information, such as pheromone trails and home-range markings (Devigne et al. 2004; Czaczkes et al. 2011; Grüter et al. 2011). Individuals may use both social and private information to help them navigate the trail system (Grüter et al. 2011; Czaczkes et al. 2013a). Social information may be deliberately signalled in a number of ways, including by direct guiding, laying trail pheromone and stridulation (Hölldobler & Wilson 1990; Roces & Hölldobler 1996; Franks & Richardson 2006). Information may also be in the form of incidental cues, such as the density of other ants on the trail, or the presence of passively deposited home-range markings (Burd & Aranwela 2003; Devigne et al. 2004; Czaczkes et al. 2013b).

An important question is whether, when making a decision, information sources and other factors are prioritised in a hierarchy, or work in concert. In situations where animals have to decide between two or more conflicting information sources or environmental effects, several options are open to them. They may choose to preferentially follow one source of information, they may take a mixed strategy, sometimes following one source and sometimes the other, or they may choose to ignore both information sources. In honeybees and many ants, when social information (pheromone trails or waggle dances) conflicts with private information (memory), private information is often prioritised (Vilela et al. 1987; Aron et al. 1988; Fourcassie & Beugnon 1988; Harrison et al. 1989; Grüter et al. 2011). In contrast, in a few ants, social information is prioritised, and when social information and private information conflict, ants preferentially follow the pheromone trails (Carthy 1951; Aron et al. 1993). This suggests a hierarchy of information sources, with private information often being prioritised over social information.

Environmental factors are a further information source known to affect ant foraging. However, unlike social and public information, these have not been well studied. Some laboratory research has investigated trail systems more representative of natural trail geometries, for example by increasing the number of bifurcations, so that the trail system has two levels of branching to give 4 end locations (Czaczkes et al. 2013a). Trail choice within this more complex trail system is not as predicted by studies using a single bifurcation, demonstrating the value of studying more complex and realistic geometries. Another example of an environmental factor affecting foraging, sometimes referred to as the ‘edge effect’, is the tendency for ants to follow walls and the edges of obstacles (Dussutour et al. 2005). Ants also have a tendency to navigate towards conspicuous objects in the environment (Graham et al. 2003).

Most studies of ant trail networks are carried out in the laboratory and use simple trail geometries and symmetrical bifurcations. However, trail bifurcation asymmetry is a potentially important environmental effect. Pharaohs ant (*Monomorium pharaonis*) foragers use unequal trail bifurcation angles to get information about their travel direction in a foraging network (Jackson et al. 2004). Studies of Argentine ants (*Linepithema humile*) show that foragers more often take the bifurcation branch that deviates less from their current route (Vittori et al. 2006; Gerbier et al. 2008). When trail pheromone is present, this environmental effect is reduced (Gerbier et al. 2008). The effect of bifurcation asymmetry on individual path choice translates to changes in group behaviour, allowing colonies to select shorter routes (Garnier et al. 2009, 2013). Studying environmental effects is important because this will help bridge the gap between laboratory studies using geometrically simple trail systems and foraging under more natural conditions (Acosta et al. 1993; Buhl et al. 2009).

The best-studied cases of the interaction of different information sources in ants come from the study of route memories interacting with trail pheromones. These two information sources have a synergistic effect on straight sections of the trail network, causing increased walking speed when the two information sources complement one another (Czaczkes et al. 2011). Moreover, the presence of trail pheromone can enhance route learning (Czaczkes et al. 2013a). Route learning in *Lasius niger* is very rapid, with 95% of foragers learning the single feeder branch at a T-bifurcation after three visits. However, when foragers face conflicting information at a T-bifurcation, memory is prioritised over trail pheromone (Grüter et al. 2011).

The few previous studies of bifurcation asymmetry on trail choice in ants have investigated only a single angle of asymmetry (Vittori et al. 2006; Gerbier et al. 2008). The aim of this study was to investigate the effects of varying branch asymmetry at a 90° bifurcation on trail choice by *L. niger* foragers and how this is influenced by the presence of trail pheromone on either the more or less deviating branch. Ants regularly forage on plants with a bifurcating structure and often form a bifurcating network of trails even in open terrain. In most laboratory studies of trail choice, environmental effects such as variation in bifurcation angle are reduced as much as possible; here, we are making them the focus of our study. Studying the role of trail bifurcation angle on ant path choice helps bridge the gap between highly artificial laboratory studies and field conditions.

## Materials and Methods

### Study Species

Workers from 4 *L. niger* colonies were collected on the University of Sussex campus and housed in plastic foraging boxes (40 × 30 × 20 cm high) coated on the inside walls with Fluon to prevent escape and with a 1-cm layer of plaster of Paris on the bottom to absorb moisture and maintain humidity. Within each foraging box was a circular plaster nest box (14 × 2 cm high). These laboratory colonies were queenless with approximately 500–1000 workers and brood. Colonies were given water *ad libitum*, and Bhatkar diet (Bhatkar & Whitcomb 1970) three times per week except in the 4 d prior to a foraging trial.

### Experiment 1: Equal Pheromone, Varying Bifurcation Asymmetry

Several weeks prior to the experiment, each colony was split into two fragments. One, dubbed the trail-laying fragment, was used to establish trails that were followed by ants of the other, the test fragment.

### Preparing the Test Colony for a Trail Choice Trial

**Fig. 1:**
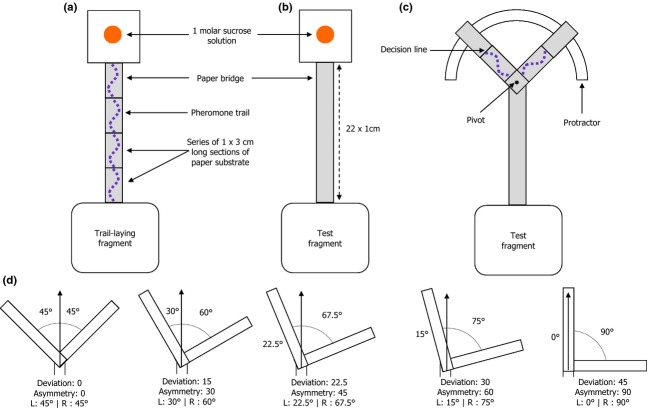
Schematic of experimental set-up (not to scale), showing the apparatus used (a) to obtain paper substrate marked with trail pheromone from the trail-laying colony, (b) to prepare the test colony for a trail choice trial by stimulating foraging and (c) to conduct a trail choice trial using the test colony, with Y-bifurcation arms covered in pheromone-marked paper obtained from (a). In this diagram, the bifurcation is set at 45:45°. The asymmetries tested are illustrated in (d). Mirrored asymmetries were also tested.

The purpose of this procedure was to initiate foraging by the test fragment and to put the foragers in the motivational state to forage (Czaczkes et al. 2014), so that naïve ants would rapidly move onto the trail and through the bifurcation when an experimental trail with Y-bifurcation was provided. To do this, the test fragment was given access to an exposed 1-cm-wide drop of 1 molar sucrose syrup via a 1 × 22-cm-long straight paper bridge (Fig.1b). Four to five foragers were allowed to reach the feeder and were marked with a dot of acrylic paint while feeding. These individuals then returned to the nest and alerted their nestmates to the presence of food. The bridge was removed before any recruits left the foraging box.

### Obtaining Pheromone-Marked Substrate from the Trail-Laying Colony

The purpose of this procedure was to obtain paper marked with trail pheromone that could be reassembled to form the branches of a Y-bifurcation, allowing foragers of the test fragment a choice between two branches equally marked with trail pheromone. Concurrently to the test fragment being stimulated to forage, the trail-laying fragment was given access to its own drop of 1 molar sucrose syrup via a straight paper bridge marked into four 1 × 3-cm-long sections (Fig.1a). An unrestricted number of foragers were allowed to walk to the feeder. On returning to the nest, many would now lay trail pheromone. *Lasius niger* have a characteristic pheromone-laying behaviour in which an individual briefly pauses, curves its abdomen down, ‘dots’ pheromone onto the substrate, lifts its abdomen and then resumes walking (Beckers et al. 1992a,b). Pheromone dots per 3-cm section were counted as they were made. When a section had 15 dots, it was covered with a piece of laminated paper to prevent further pheromone accumulation. When all sections had 15 dots, the paper was removed and cut into four 1 × 3-cm pieces. Two of these were placed in a freezer at −20°C to delay pheromone evaporation. The other two were immediately used to make a Y-bifurcation (Fig.1c).

### Trail Choice Trial Procedure

This procedure quantified how different levels of bifurcation asymmetry affect naïve foragers, either with equal pheromone on each branch (experiment 1) or with pheromone present on only one branch (experiment 2). In a trial, the test colony was given a bridge attached to a 90° Y-bifurcation with a pivot (Fig.1c).

The branch taken by each ant at the bifurcation was recorded. An individual was said to have made a choice when the first antenna crossed a marked ‘decision’ line, 3 cm past the bifurcation point and where the pheromone-marked paper ended. The ant was then removed using an aspirator and returned to the foraging box at the end of the trial. If a marked ant was observed, it was removed without any data being taken. This ensured that only choices of naïve foragers were recorded. When ten individuals had passed through the Y-bifurcation, the V part was swivelled on its pivot to another angle treatment. Five different treatments, with the V part of the bifurcation deviating between 0° and 90° from having 45° to both left and right, to give asymmetries of 0°, 30°, 45°, 60° and 90° (Fig.1d), were tested in a random order. Data were collected in this manner until 45 min after the first pheromone dot had been laid. The two 3-cm strips were then replaced with the fresh pheromone strips being kept in the freezer. This was performed to ensure that all data were collected while a strong pheromone trail was still present. A single dot of *L. niger* trail pheromone has been estimated to become undetectable in 47 min at room temperature, and established trails have been estimated to remain detectable for 24 h or longer (Beckers et al. 1993; Evison et al. 2008). Thus, a time limit of 45 min is conservative and ensures that by the end of the 45 min, the pheromone is still detectable. Data collection then resumed for a further 45 min. At the end of the trial, the bridge was removed, both colonies were fed, and the apparatus was cleaned with ethanol (plastic components) or replaced (paper components).

### Experiment 2: Only One Arm of the Y-Maze Marked with Pheromone

Methods for experiment 2 were the same as for experiment 1, except that laminated paper overlays were used throughout the trail-laying stage to prevent any pheromone laying on 2 of the 1 × 3-cm sections. When 2 strips were used to make the head of the Y-bifurcation, a pheromone-marked strip on one side was paired with a pheromone-free strip on the other. The pheromone-marked side (i.e. whether it was the left or right branch of the bifurcation) was alternated between trials, to ensure that if the ants had any systematic left vs. right preference, it would not bias the overall results.

### Statistical Analysis

Data were analysed in R v. 2.15.1 (R Development Core Team 2009). The proportion of ants choosing the less deviating branch (experiments 1 and 2) or choosing the pheromone-marked branch (experiment 2) was modelled using eneralised linear mixed-effect models (GLMMs), using the LMER function in the LME4 package (Bates et al. 2007). GLMMs were used as they are capable of handling random effects. In our experiment, ‘colony’ was always added as a random effect which was free to vary in both slope and intercept (Zuur et al. 2009). Following Forstmeier & Schielzeth (2011) (see also Orelien & Edwards 2008; Symonds & Moussalli 2010), model selection was conducted by including only fixed effects and interactions we had an *a priori* reason for including, namely asymmetry angle, the location of the pheromone-marked trail (was it the more deviating or less deviating branch) and the interaction between the two. A binomial distribution family was modelled using the logit function. Simple comparison of choice data to a null hypothesis of random choice was performed using sign tests. All p-values are corrected for multiple testing using the Benjamini–Hochberg method (Benjamini & Hochberg 1995).

## Results

### Experiment 1: Equal Pheromone, Varying Bifurcation Asymmetry

**Fig. 2:**
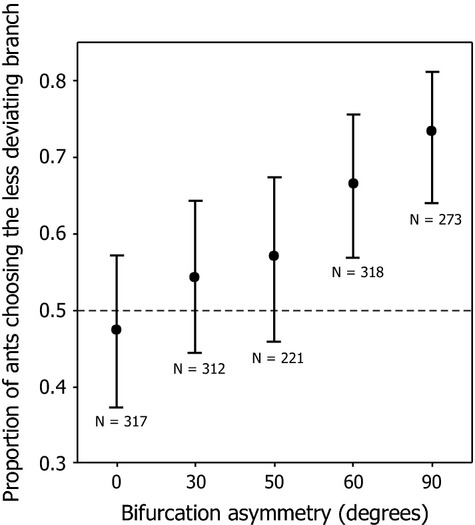
Proportion of ants choosing the less deviating branch on a Y-maze in which both arms are marked with trail pheromone. Ants preferentially follow the less deviating branch, and this effect is strengthened as bifurcation asymmetry increases. An asymmetry of 45° means that the two branches were at 0°:90° or 90°:0° from the stem of the Y, 30° means that the two branches were at 30°:60° or 60°:30°, etc. (Fig.1d). At 0° asymmetry (branches at 45°:45°), the right branch was arbitrarily chosen to represent the straighter branch. Dots are means, and whiskers are 95% confidence intervals for the means, as estimated by the statistical model.

The results of experiment 1 show that as asymmetry increases, the ants were more likely to choose the less deviating branch (GLMM, Z = 6.971, p < 0.00001, Fig.2). When the bifurcation was symmetrical (45° to both left and right; asymmetry 0°), 47% of ants chose the right branch. At the maximum bifurcation asymmetry of 45° (the more deviating branch being at 90° from the Y-maze stem and the less deviating branch continuing directly onwards from the stem), 73% of ants chose the less deviating branch.

### Experiment 2: Only One Arm of the Y-Maze Marked with Pheromone

**Fig. 3:**
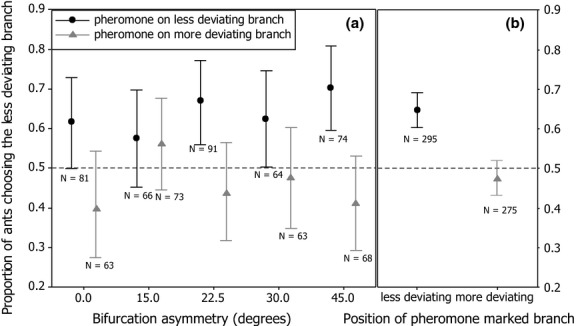
Proportion of ants choosing the less deviating branch on a Y-maze in which one arm is marked with trail pheromone. Ants are more likely to follow the less deviating branch if it is also marked with trail pheromone and less likely to follow it if the opposite branch is marked with trail pheromone (a&b). However, the level of bifurcation asymmetry does not affect the likelihood of ants to choose the less deviating branch when pheromone is present on only one branch (a). Symbols are means, whiskers are 95% confidence intervals for the means, as estimated by the statistical model.

Over all bifurcation angles, ants were more likely to choose the pheromone-marked branch (two-tailed sign test, N = 570, 336 ants chose the pheromone-marked branch, p < 0.0001). Pooling data from trials where either the more or less deviating branch was marked with trail pheromone, ants were more likely to choose the less deviating branch (two-tailed sign test, N = 570, 321 ants chose the less deviating branch, p = 0.004). There was no significant effect of increasing branch asymmetry on its own (GLMM, Z = 0.717, p = 0.473) or in interaction with the presence of the pheromone-marked branch (GLMM, Z = −1.056, p = 0.291) on the probability of ants choosing the less deviating branch (Fig.3a). However, the location of the pheromone did have a significant effect. Ants were more likely to choose the less deviating branch if it was marked with trail pheromone (GLMM, Z = −4.919, p < 0.0001, Fig.3b), and similarly, the pheromone-marked branch was more likely to be chosen by ants if it was also the less deviating branch (GLMM, Z = 2.935, p = 0.0033).

## Discussion

A basic goal of this project was to explore the effort of path bifurcation on branch choice. Experiment 1 shows that asymmetry at a trail bifurcation has a significant effect on trail choice by naïve outgoing *L. niger* foragers, with ants preferring the less deviating branch. In experiment 2, when trail pheromone is only present on one of the two branches, ants still prefer the less deviating branch, but the preference for the less deviating branch did not increase as the asymmetry increased (Fig.3a). The preference of ants for less deviating paths, and the interaction of pheromone trails with this preference, should have consequences for colony-level path selection, allowing ant colonies to efficiently choose shorter paths to food sources (Garnier et al. 2013; Vela-Pérez et al. 2013). In terms of effect strengths, trail asymmetry is similar or somewhat weaker than the effect of trail pheromone [4–23% above random choice vs. 11–34% in this experiment and in Grüter et al. (2011)] and clearly weaker than the effect of route memory [25–45% above random choice in Grüter et al. (2011)].

### Multiple Factors Influencing Branch Choice

One goal of this project was to elucidate how information use is prioritised during branch choice in ants. Experiment 2, in which only one of the bifurcation braches was marked by trail pheromone, possesses two factors that can affect trail choice, social information in the form of trail pheromone and the environmental effect of trail asymmetry. We find no clear hierarchy between these information sources. Foragers do not seem to prioritise either trail pheromones or trail straightness, as when the two conflict at a bifurcation, the two branches are chosen with almost equal probability. While the presence of an information source hierarchy is not supported, it is not clear exactly how the two information sources are interacting. The ants may either be choosing randomly between the two branches or the two factors both have an effect that cancels each other out. In addition, the presence of pheromone on one branch seems to remove the graded effect of trail asymmetry seen when pheromone is present on both branches (experiment 1) and even has a subtractive effect.

As more research is carried out into the how multiple factors affect decision-making during foraging and navigation, it seems that interactive effects of some kind are the rule (Wetterer et al. 1992; Franks et al. 2007; Czaczkes et al. 2011, 2013a). The concept of a hierarchy of information sources, with higher sources used exclusively until they become unavailable, appears to be only rarely the case (Rosengren & Fortelius 1986; Vilela et al. 1987; Banks & Srygley 2003). However, it seems that foragers utilise different information sources depending on what is available and can rely on a subset of information if need be (Dyer & Could 1983; Vilela et al. 1987; Chittka et al. 1999). The way in which ants use and prioritise information when choosing a path seems to be complex and is still not fully understood, as evidenced from the puzzling effect seen in experiment 2. Why does the graded effect, but not the main effect, of trail asymmetry disappear when pheromone trails become more informative? It is clear that in this case, there is not a simple additive, synergistic or prioritisation effect.

The way in which different factors influence decision-making, and the weight given to different sources of information, is also known to vary depending on the situation (Czaczkes et al. 2014). Forager honey bees continue to use private information for a long time to after a feeding site has been made unrewarding, but are eventually able to switch to using social information, in the form of the waggle dance (Al Toufailia et al. 2013; Grüter et al. 2013). The relative importance of a pheromone trail might be lower for ants returning to the nest, which may affect how foragers use or prioritise various information sources and environmental effects. Foragers make consistently different choices at bifurcations depending on whether they are outgoing or returning to the nest (Jander 1990). This is because the goal of the forager in each situation is different. Differing goals between outgoing and returning ants may result if a different response to branch asymmetry or a different balance between branch asymmetry and trail pheromone effects. Returning ants will also always have some memory of their goal.

### Application to Field Conditions

Another goal of this study was to help bridge the gap between results from highly simplified laboratory studies and the field, where environmental effects vary greatly and are likely to be more important. Overall, our results show that the environmental effect of trail asymmetry at a bifurcation has considerable influence on ant foraging. In the field, environmental effects may affect the pattern of exploration of virgin territory (Jander 1990) or how trails that have been disrupted, for example by an obstacle landing on a pheromone trail, are re-established. The results of this study suggest that field environmental effects will lead to a preference for some directions over others when ants are establishing or strengthening a trail. Most obviously, by favouring branches that are less deviating, outgoing ants are more likely to discover food sources reached via straighter paths. Straight trails are less likely to cross other trails and should result in a more efficient exploration of space.

The environmental effect we studied will not normally act in isolation. For large or replenishing food sources, it will act in concert with trail deposition behaviour and individual memory. In terms of trail pheromone deposition, branches and paths whose direction is closer to a direct route to or from the nest are more heavily marked (Beckers et al. 1992a,b). Thus, not only are resources more likely to be discovered in locations where environmental effects give straighter paths, the trails to these resources will be strengthened faster or more, which would tend to prolong the effect. In *L. niger,* route memory overrides trail pheromones at a bifurcation (Grüter et al. 2011). Therefore, an environmental bias favouring a straighter path or branch could probably be overcome by route memory. This hypothesis remains to be tested. However, route memory formation is also strongly affected by path layout (Czaczkes et al. 2013a), and *L. niger* foragers are quicker to memorise routes which require repeated (e.g. left then left) vs. alternating branch choices (e.g. left then right). This could result in colonies exploiting some food sources rather than others, even if the quality and distance from the nest of the food sources are identical. The environmental effect on route memory is so strong that even if a food source at the end of a difficult-to-learn route (e.g., left then right) is discovered first, colonies will nonetheless quickly focus foraging on an easier-to-learn route when food is also provided at this location (C. Grüter & F. L. W. Ratnieks, unpubl. data). Overall, environmental effects may well have a very strong influence in the field and may cause foraging to be unevenly distributed in a colony's territory, with their influence affecting not just naïve ants or the initial discovery of a feeding location. Further studies linking laboratory results to the foraging behaviour and ecology of ant colonies in the field are needed.

## References

[b1] Acosta FJ, López F, Serrano JM (1993). Branching angles of ant trunk trails as an optimization cue. J. Theor. Biol.

[b2] Al Toufailia H, Couvillon MJ, Ratnieks FLW, Grüter C (2013). Honey bee waggle dance communication: signal meaning and signal noise affect dance follower behaviour. Behav. Ecol. Sociobiol.

[b3] Aron S, Deneubourg JL, Pasteels JM (1988). Visual cues and trail-following idiosyncrasy in *leptothorax unifasciatus*: An orientation process during foraging. Insectes Soc.

[b4] Aron S, Beckers R, Deneubourg J, Pasteels JM (1993). Memory and chemical communication the orientation of two mass-recruiting ant species. Insectes Soc.

[b5] Banks AN, Srygley RB (2003). Orientation by magnetic field in leaf-cutter ants, *Atta colombica* (Hymenoptera: Formicidae). Ethology.

[b6] Bates D, Sarkar D, Bates MD, Matrix LT (2007).

[b7] Beckers R, Deneubourg J, Goss S (1992a). Trail laying behaviour during food recruitment in the ant *Lasius niger* (L.). Insectes Soc.

[b8] Beckers R, Deneubourg L, Goss S (1992b). Trails and U-turns in the selection of a path by the Ant *Lasius niger*. J. Theor. Biol.

[b9] Beckers R, Deneubourg JL, Goss S (1993). Modulation of trail laying in the ant *Lasius niger* (Hymenoptera: Formicidae) and its role in the collective selection of a food source. J. Insect Behav.

[b10] Benjamini Y, Hochberg Y (1995). Controlling the false discovery rate: a practical and powerful approach to multiple testing. J. R. Stat. Soc. Ser. B Methodol.

[b11] Bhatkar A, Whitcomb WH (1970). Artificial diet for rearing various species of Ants. Fla. Entomol.

[b12] Buhl J, Hicks K, Miller ER, Persey S, Alinvi O, Sumpter DJT (2009). Shape and efficiency of wood ant foraging networks. Behav. Ecol. Sociobiol.

[b13] Burd M, Aranwela N (2003). Head-on encounter rates and walking speed of foragers in leaf-cutting ant traffic. Insectes Soc.

[b14] Carthy JD (1951). The orientation of two allied species of British Ant, II. Odour trail laying and following in *Acanthomyops (Lasius) fuliginosus*. Behaviour.

[b15] Chittka L, Williams NM, Rasmussen H, Thomson JD (1999). Navigation without vision: bumblebee orientation in complete darkness. Proc. R. Soc. Lond. B Biol. Sci.

[b16] Czaczkes TJ, Grüter C, Jones SM, Ratnieks FLW (2011). Synergy between social and private information increases foraging efficiency in ants. Biol. Lett.

[b17] Czaczkes TJ, Grüter C, Ratnieks FLW (2013a). Ant foraging on complex trails: route learning and the role of trail pheromones in *Lasius niger*. J. Exp. Biol.

[b18] Czaczkes TJ, Grüter C, Ratnieks FLW (2013b). Negative feedback in ants: crowding results in less trail pheromone deposition. J. R. Soc. Interface.

[b501] Czaczkes TJ, Schlosser L, Heinze J, Witte V (2014). Ants use directionless odour cues to recall odour-associated locations. Behav. Ecol. Sociobiol.

[b19] Devigne C, Renon A, Detrain C (2004). Out of sight but not out of mind: modulation of recruitment according to home range marking in ants. Anim. Behav.

[b20] Dussutour A, Deneubourg J-L, Fourcassié V (2005). Amplification of individual preferences in a social context: the case of wall-following in ants. Proc. R. Soc. B Biol. Sci.

[b21] Dyer FC, Could JL (1983). Honey Bee Navigation: The honey bee's ability to find its way depends on a hierarchy of sophisticated orientation mechanisms. Am. Sci.

[b22] Evison SEF, Petchey OL, Beckerman AP, Ratnieks FLW (2008). Combined use of pheromone trails and visual landmarks by the common garden ant *Lasius niger*. Behav. Ecol. Sociobiol.

[b23] Forstmeier W, Schielzeth H (2011). Cryptic multiple hypotheses testing in linear models: overestimated effect sizes and the winner's curse. Behav. Ecol. Sociobiol.

[b24] Fourcassie V, Beugnon G (1988). How do red wood ants orient when foraging in a three dimensional system? I. Laboratory experiments. Insectes Soc.

[b25] Franks NR, Richardson T (2006). Teaching in tandem-running ants. Nature.

[b26] Franks NR, Hooper JW, Dornhaus A, Aukett PJ, Hayward AL, Berghoff SM (2007). Reconnaissance and Latent Learning in Ants. Proc. R. Soc. B Biol. Sci.

[b27] Garnier S, Guérécheau A, Combe M, Fourcassié V, Theraulaz G (2009). Path selection and foraging efficiency in Argentine ant transport networks. Behav. Ecol. Sociobiol.

[b28] Garnier S, Combe M, Jost C, Theraulaz G (2013). Do ants need to estimate the geometrical properties of trail bifurcations to find an efficient route? A Swarm robotics test bed. PLoS Comput. Biol.

[b29] Gerbier G, Garnier S, Rieu C, Theraulaz G, Fourcassié V (2008). Are ants sensitive to the geometry of tunnel bifurcation?. Anim. Cogn.

[b30] Graham P, Fauria K, Collett TS (2003). The influence of beacon-aiming on the routes of wood ants. J. Exp. Biol.

[b31] Grüter C, Czaczkes TJ, Ratnieks FLW (2011). Decision making in ant foragers (*Lasius niger*) facing conflicting private and social information. Behav. Ecol. Sociobiol.

[b32] Grüter C, Segers FHID, Ratnieks FLW (2013). Social learning strategies in honeybee foragers: do the costs of using private information affect the use of social information?. Anim. Behav.

[b33] Harrison JF, Fewell JH, Stiller TM, Breed MD (1989). Effects of experience on use of orientation cues in the giant tropical ant. Anim. Behav.

[b34] Hölldobler B, Wilson EO (1990). The Ants.

[b35] Jackson DE, Holcombe M, Ratnieks FLW (2004). Trail geometry gives polarity to ant foraging networks. Nature.

[b36] Jander R (1990). Arboreal search in ants: search on branches (Hymenoptera: Formicidae). J. Insect Behav.

[b37] Orelien JG, Edwards LJ (2008). Fixed-effect variable selection in linear mixed models using statistics. Comput. Stat. Data Anal.

[b38] R Development Core Team (2009). R: A Language and Environment for Statistical Computing.

[b39] Roces F, Hölldobler B (1996). Use of stridulation in foraging leaf-cutting ants: mechanical support during cutting or short-range recruitment signal?. Behav. Ecol. Sociobiol.

[b40] Rosengren R, Fortelius W (1986). Ortstreue in foraging ants of the *Formica rufa* group — Hierarchy of orienting cues and long-term memory. Insectes Soc.

[b41] Symonds MRE, Moussalli A (2010). A brief guide to model selection, multimodel inference and model averaging in behavioural ecology using Akaike's information criterion. Behav. Ecol. Sociobiol.

[b42] Vela-Pérez M, Fontelos MA, Velázquez JJL (2013). Ant foraging and geodesic paths in labyrinths: analytical and computational results. J. Theor. Biol.

[b43] Vilela EF, Jaffé K, Howse PE (1987). Orientation in leaf-cutting ants (Formicidae: Attini). Anim. Behav.

[b44] Vittori K, Talbot G, Gautrais J, Fourcassie V, Arauījo AFR, Theraulaz G (2006). Path efficiency of ant foraging trails in an artificial network. J. Theor. Biol.

[b45] Wetterer JK, Shafir S, Morrison L, Lips K, Gilbert G, Cipollini M, Blaney C (1992). On- and off-trail Orientation in the leaf-cutting Ant, *Atta cephalotes* (L.) (Hymenoptera: Formicidae). J. Kans. Entomol. Soc.

[b46] Zuur AF, Ieno EN, Walker NJ, Saveliev AA, Smith GM (2009). Mixed Effects Models and Extensions in Ecology with R.

